# Superfluidity of Dipolar Excitons in a Double Layer of *α* − *T*_3_ with a Mass Term

**DOI:** 10.3390/nano12091437

**Published:** 2022-04-22

**Authors:** Oleg L. Berman, Godfrey Gumbs, Gabriel P. Martins, Paula Fekete

**Affiliations:** 1 Physics Department, New York City College of Technology, City University of New York, New York, NY 11201, USA; gpimentamartins@gradcenter.cuny.edu; 2 The Graduate School and University Center, City University of New York, New York, NY 10016, USA; ggumbs@hunter.cuny.edu; 3 Department of Physics and Astronomy, Hunter College, City University of New York, New York, NY 10065, USA; 4 Donastia International Physics Center (DIPC), P de Manuel Lardizabal, 4, 20018 San Sebastian, Spain; 5 US Military Academy at West Point, 606 Thayer Road, West Point, NY 10996, USA; paula.fekete@westpoint.edu

**Keywords:** Bose-Einstein condensation, superfluidity, dipolar exitons

## Abstract

We predict Bose-Einstein condensation and superfluidity of dipolar excitons, formed by electron-hole pairs in spatially separated gapped hexagonal $\alpha -T_{3}$ (GHAT3) layers. In the $\alpha -T_{3}$ model, the AB-honeycomb lattice structure is supplemented with C atoms located at the centers of the hexagons in the lattice. We considered the $\alpha -T_{3}$ model in the presence of a mass term which opens a gap in the energy-dispersive spectrum. The gap opening mass term, caused by a weak magnetic field, plays the role of Zeeman splitting at low magnetic fields for this pseudospin-1 system. The band structure of GHAT3 monolayers leads to the formation of two distinct types of excitons in the GHAT3 double layer. We consider two types of dipolar excitons in double-layer GHAT3: (a) “A excitons”, which are bound states of electrons in the conduction band (CB) and holes in the intermediate band (IB), and (b) “B excitons”, which are bound states of electrons in the CB and holes in the valence band (VB). The binding energy of A and B dipolar excitons is calculated. For a two-component weakly interacting Bose gas of dipolar excitons in a GHAT3 double layer, we obtain the energy dispersion of collective excitations, the sound velocity, the superfluid density, and the mean-field critical temperature $T_{c}$ for superfluidity.

## 1. Introduction

The many-particle systems of dipolar (indirect) excitons, formed by spatially separated electrons and holes, in semiconductor coupled quantum wells (CQWs) and novel two-dimensional (2D) materials have been the subject of numerous experimental and theoretical studies. These systems are attractive in large part due to the possibility of Bose-Einstein condensation (BEC) and superfluidity of dipolar excitons, which can be observed as persistent electrical currents in each quantum well, and also through coherent optical properties [1,2,3,4,5]. Recent progress in theoretical and experimental studies of BEC and superfluidity of dipolar excitons in CQWs have been reviewed in [6]. Electron-hole superfluidity in double layers can occur not only in the BEC regime, but also in the Bardeen-Cooper-Schrieffer (BCS)-BEC crossover regime [7].

A number of experimental and theoretical investigations have been devoted to the BEC of electron-hole pairs, formed by spatially separated electrons and holes in a double layer formed by parallel graphene layers. These investigations were reported in [8,9,10,11,12,13]. Both BEC and superfluidity of dipolar excitons in double layers of transition-metal dichalcogenides (TMDCs) [14,15,16,17,18] and phosphorene [19,20] have been discussed, because the exciton binding energies in novel 2D semiconductors are quite large. Possible BEC in a long-lived dark spin state of 2D dipolar excitons has been experimentally observed for GaAs/AlGaAs semiconductor CQWs [21].

Recently, the electronic properties of the $\alpha -T_{3}$ lattice have been the subject of the intensive theoretical and experimental investigations due to its surprising fundamental physical properties as well as its promising applications in solid-state devices [22,23,24,25,26,27,28,29,30,31,32,33,34,35]. For a review of artificial flat band systems, see [36]. Raoux, et al. [22] proposed that an $\alpha -T_{3}$ lattice could be assembled from cold fermionic atoms confined to an optical lattice by means of three pairs of laser beams for the optical dice lattice (α=1) [37]. This structure consists of an AB-honeycomb lattice (the rim) like that in graphene which is combined with C atoms at the center/hub of each hexagon. A parameter α represents the ratio of the hopping integral between the rim and the hub to that around the rim of the hexagonal lattice. By dephasing one of the three pairs of laser beams, one could vary the parameter 0≤α=tanφ≤1. Optically induced dressed states [38], and their tunneling, transport [33,39], and collective properties [40], as well as $\alpha -T_{3}$ based nanoribbons [41] have been analyzed. The BEC and superfluidity of dipolar magnetoexcitons in $\alpha -T_{3}$ double layers in a strong uniform perpendicular magnetic field were proposed in [42].

We present the conditions for BEC and superfluidity of a two-component weakly interacting Bose gas of dipolar excitons, formed by electron-hole pairs in spatially separated GHAT3 layers. An applied weak magnetic field to this pseudospin-1 monolayer system results in a Zeeman-type splitting of the energy subbands [43]. This dispersion relation consists of three bands: CB, IB, and VB. We consider two types of dipolar excitons in a double-layer of GHAT3: (a) “A excitons”, formed as bound states of electrons in CB and holes in IB, and (b) “B excitons”, formed as bound states of electrons in CB and holes in VB. The binding energy of A and B dipolar excitons is calculated. For a two-component weakly interacting Bose gas of dipolar excitons in a GHAT3 double layer, we obtain the energy dispersion of collective excitations, the sound velocity, the superfluid density, and the mean-field critical temperature $T_{c}$ for superfluidity.

Our paper is organized in the following way. In Section 2, the two-body problem for an electron and a hole, spatially separated in two parallel GHAT3 monolayers, is formulated, and the effective masses and binding energies are obtained for two types of dipolar excitons. The spectrum of collective excitations and the sound velocity for the two-component weakly interacting Bose gas of dipolar excitons in the double layer of GHAT3 are derived in Section 3. In Section 4 the superfluidity of the weakly interacting Bose gas of dipolar excitons in the double layer of GHAT3 is predicted, and the mean-field critical temperature of the phase transition is obtained. The results of our calculations are discussed in Section 5. In Section 6 our conclusions are reported.

## 2. Dipolar Excitons in a Double Layer of $\alpha -T_{3}$ with a Mass Term

We will consider charge carriers in the conduction band, valence band, and the intermediate band, which corresponds to the flat band in an $\alpha -T_{3}$ layer without a mass term. In the presence of a weak magnetic field, the low-energy Hamiltonian of the charge carriers in a GHAT3 monolayer at the K and K’ points are given by [43]

(1) $$\begin{array}{c} {\hat{H}}_{\lambda }=\left(\begin{array}{ccc} \Delta & f(k)\cos\phi & 0\\ f^{*}\left(k\right)\cos\phi & 0 & f(k)\sin\phi \\ 0 & f^{*}\left(k\right)\sin\phi & -\Delta \end{array}\right), \end{array}$$

where the origin in k-space is defined to be around the K point, $k=(k_{x},k_{y})$ and $\tan\theta_{k}=k_{y}/k_{x}$, $\phi =\tan^{-1}\alpha$, $f\left(k\right)=\hbar v_{F}\left(\lambda k_{x}-ik_{y}\right)=\lambda \hbar v_{F}ke^{-i\lambda \theta_{k}}$, with λ=±1 being the valley index at the K and K’ points, 2Δ is the gap in the energy spectrum of a GHAT3 layer due to the mass term in the Hamiltonian. In an $\alpha -T_{3}$ layer honeycomb lattice, there is an added fermionic hub atom C at the center of each hexagon. Let the hopping integral be $t_{1}$ between the hub atom and either an A or B atom on the rim and $t_{2}$ between nearest neighbors on the rim of the hexagon. The ratio of these two nearest neighbor hopping terms is denoted as $t_{2}/t_{1}=\alpha$, where the parameter α satisfies 0≤α≤1. The largest value when α is 1 is for the dice lattice, whereas its value of 0 corresponds to graphene for decoupled hub from rim atoms [43].

At small momenta near K and K’ points, the dispersion for the charge carriers in the conduction band $\epsilon_{CB}\left(k\right)$ is given by the relation [43]

(2) $$\begin{array}{c} \epsilon_{CB}\left(k\right)\approx \Delta +\frac{\hbar^{2}k^{2}}{2m_{CB}}, \end{array}$$

where k=p/ħ and p are the wave vector and momentum of a quasiparticle, $m_{CB}$ is the effective mass of the charge carriers in the conduction band, given by

(3) $$\begin{array}{c} m_{CB}=\frac{\left(1+\alpha^{2}\right)\Delta }{2v_{F}^{2}}, \end{array}$$

where $v_{F}$ is the Fermi velocity in a GHAT3 layer, and $\varphi =\tan^{-1}\alpha$ [43]. At small momenta near K and K’ points, the dispersion for the charge carriers in the valence band $\epsilon_{VB}\left(k\right)$ is given by the relation [43]

(4) $$\begin{array}{c} \epsilon_{VB}\left(k\right)\approx -\Delta -\frac{\hbar^{2}k^{2}}{2m_{VB}}, \end{array}$$

with $m_{VB}$ the effective mass of the charge carriers in the valence band, given by

(5) $$\begin{array}{c} m_{VB}=\frac{\left(1+\alpha^{2}\right)\Delta }{2v_{F}^{2}\alpha^{2}}. \end{array}$$

At small momenta near K and K’ points, the dispersion for the charge carriers in the intermediate band, corresponding to the flat band in an $\alpha -T_{3}$ layer without a mass term, $\epsilon_{IB}\left(k\right)$ is given by the relation [43]

(6) $$\begin{array}{c} \epsilon_{IB}\left(k\right)\approx -\frac{\hbar^{2}k^{2}}{2m_{IB}}, \end{array}$$

where $m_{IB}$ is the effective mass of the charge carriers in the intermediate band, given by

(7) $$\begin{array}{c} m_{IB}=\frac{\left(1+\alpha^{2}\right)\Delta }{2v_{F}^{2}\left(1-\alpha^{2}\right)}. \end{array}$$

It is worth noting that there are spin degeneracy and valley degeneracy for the energy of the charge carriers in a GHAT3 layer.

**Figure 1:**
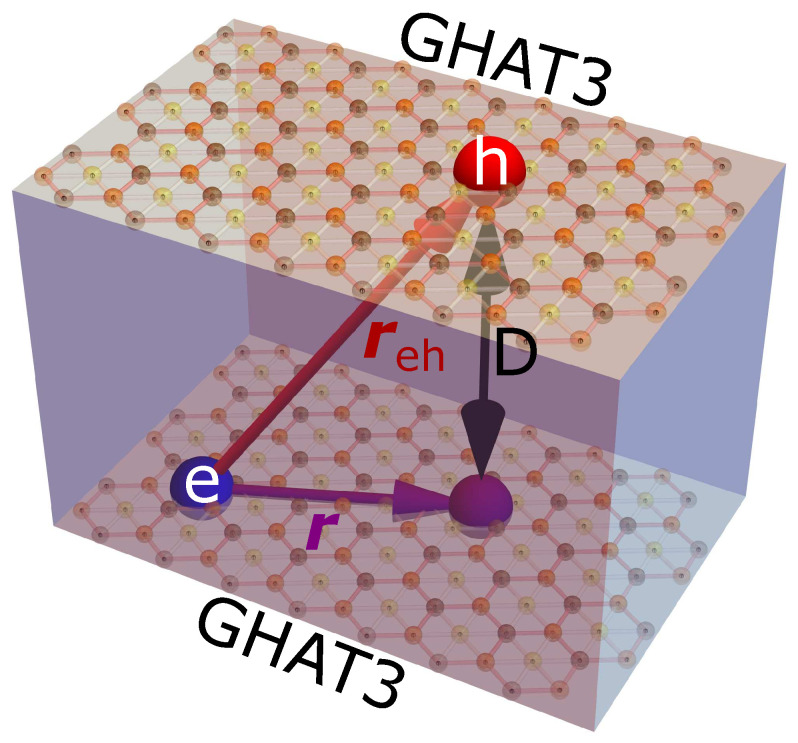
Schematic illustration of a dipolar excitonin a pair of GHAT3 double layers embedded in an insulating material.

In the system under consideration in this paper, electrons are confined in a 2D GHAT3 monolayer, while an equal number of positive holes are located in a parallel GHAT3 monolayer at a distance *D* away as demonstrated in Figure 1. This electron-hole system in two parallel GHAT3 layers is treated as a 2D system without interlayer hopping. Due to the absence of tunneling of electrons and holes between different GHAT3 monolayers, electron-hole recombination is suppressed by a dielectric barrier with dielectric constant $\epsilon_{d}$ that separates the GHAT3 monolayers. Therefore, the dipolar excitons, formed by electrons and holes, located in two different GHAT3 monolayers, have a longer lifetime than direct excitons. The electron and hole are attracted via electromagnetic interaction $V(r_{eh}),$ where $r_{eh}$ is the distance between the electron and hole, and they could form a bound state, i.e., an exciton, in three-dimensional (3D) space. Therefore, to determine the binding energy of the exciton a two-body problem in restricted 3D space has to be solved. However, if one projects the electron position vector onto the GHAT3 plane with holes and replaces the relative coordinate vector $r_{eh}$ by its projection r on this plane, the potential $V(r_{eh})$ may be expressed as $V\left(r_{eh}\right)=V\left(\sqrt{r^{2}+D^{2}}\right),$ where *r* is the relative distance between the hole and the projection of the electron position vector onto the GHAT3 plane with holes. A schematic illustration of the dipolar exciton in a GHAT3 double layer is presented in Figure 1. By introducing in-plane coordinates $r_{1}=\left(x_{1},y_{1}\right)$ and $r_{2}=\left(x_{2},y_{2}\right)$ for the electron and the projection vector of the hole, respectively (where r=$r_{1}-r_{2}$), the dipolar exciton can be described by employing a two-body 2D Schrödinger equation with potential $V(\sqrt{r^{2}+D^{2}})$. So that the restricted 3D two-body problem can be reduced to a 2D two-body problem on a GHAT3 layer with the holes.

The dipolar excitons with spatially separated electrons and holes in two parallel GHAT3 monolayers can be created by laser pumping with an applied external voltage. While an electron in the conduction band and a hole in the valence or intermediate band are excited due to absorption of a photon, voltages are applied with opposite signs to confine electrons on one layer and holes on another so that dipoles point in one direction only.

In our case, “both” the energy bands and the exciton modes referred to the K-point, not one to the Γ point and the other to the K point. We note that in the dispersion equations appearing in [43,44,45,46] the origin of the k-space was specified to be around the K point, (and not the Γ point) as did several authors investigating $\alpha -T_{3}$. So, our choice of origin not being the center of the Brillouin zone has precedence. For graphene, the plasmon dispersion relation and low-energy bands, presented by [47] were both consistently measured from the K point taken as the origin and not the center of the Brillouin zone.

We consider excitons, formed by an electron and a hole from the same valley, because an electron and a hole from different valleys cannot be excited by absorption of photon due to conservation of momentum. The reason is that photons carry momenta much smaller than the difference between K and K’ in reciprocal space.

The effective Hamiltonian of an electron and a hole, spatially separated in two parallel GHAT3 monolayers with the interlayer distance *D* has the following form

(8) $${\hat{H}}_{ex}=-\frac{\hbar^{2}}{2m_{e}}\Delta_{r_{1}}-\frac{\hbar^{2}}{2m_{h}}\Delta_{r_{2}}+V\left(r\right),$$

where $\Delta_{r_{1}}$ and $\Delta_{r_{2}}$ are the Laplacian operators with respect to the components of the vectors $r_{1}$ and $r_{2}$, respectively, and $m_{e}$ and $m_{h}$ are the effective masses of the electron and hole, respectively. For CV excitons $m_{e}=m_{CB}$ and $m_{h}=m_{VB}$; and for CI excitons $m_{e}=m_{CB}$ and $m_{h}=m_{IB}$, where $m_{CB}$, $m_{VB}$, and $m_{IB}$ are given by Equations (3), (5) and (7), correspondingly. The problem of the in-plane motion of an interacting electron and hole forming the exciton in a GHAT3 double layer can be reduced to that of one particle with the reduced mass $\mu =m_{e}m_{h}/\left(m_{e}+m_{h}\right)$ in a V(r) potential and motion of the center-of-mass of the exciton with the mass $M=m_{e}+m_{h}$. We introduce the coordinates of the center-of-mass R of an exciton and the coordinate of the relative motion r of an electron and hole as $R=\left(m_{e}r_{1}+m_{h}r_{2}\right)/\left(m_{e}+m_{h}\right)$ and $r=r_{1}-r_{2}$, correspondingly. The Hamiltonian ${\hat{H}}_{ex}$ can be represented in the form: ${\hat{H}}_{ex}={\hat{H}}_{R}+{\hat{H}}_{r}$, where the Hamiltonian of the motion of the center-of-mass is ${\hat{H}}_{R}$ and that of the relative motion of electron and a hole is ${\hat{H}}_{r}$. The solution of the Schrödinger equation for the center-of-mass of an exciton ${\hat{H}}_{R}\psi \left(R\right)=E\psi \left(R\right)$ is the plane wave $\psi \left(R\right)=e^{iP\cdot R/\hbar }$ with the quadratic energy spectrum $E=P^{2}/\left(2M\right)$, where P is the momentum of the center-of-mass of an exciton.

We consider electrons and holes to be located in GHAT3 parallel layers, embedded in a dielectric with the dielectric constant $\epsilon_{d}$. The potential energy of electron-hole Coulomb attraction is

(9) $$\begin{array}{c} V\left(r\right)=-\frac{\kappa e^{2}}{\epsilon_{d}\sqrt{r^{2}+D^{2}}}\;, \end{array}$$

where $\kappa =9\times 10^{9}\;N\times m^{2}/C^{2}$, $\epsilon_{d}$ is the dielectric constant of the insulator (SiO${}_{2}$ or *h*-BN), surrounding the electron and hole GHAT3 monolayers, forming the double layer. For the *h*-BN barrier we substitute the dielectric constant $\epsilon_{d}=4.89$, while for the SiO${}_{2}$ barrier we substitute the dielectric constant $\epsilon_{d}=4.50$. For *h*-BN insulating layers, $\epsilon_{d}=4.89$ is the effective dielectric constant, defined as $\epsilon_{d}=\sqrt{\varepsilon^{⊥}}\sqrt{\varepsilon^{\|}}$ [14], where $\varepsilon^{⊥}=6.71$ and $\varepsilon^{\|}=3.56$ are the components of the dielectric tensor for *h*-BN [48]. Assuming r≪D, we approximate V(r) by the first two terms of the Taylor series and obtain

(10) $$\begin{array}{c} V\left(r\right)=-V_{0}+\gamma r^{2},\;\;\;\mathrm{where}\;V_{0}=\frac{\kappa e^{2}}{\epsilon_{d}D},\;\;\;\;\;\;\;\;\gamma =\frac{\kappa e^{2}}{2\epsilon_{d}D^{3}}. \end{array}$$

The similar approach has been applied for excitons in TMDC double layers [16,17]. The solution of the Schrödinger equation for the relative motion of an electron and a hole ${\hat{H}}_{r}\Psi \left(r\right)=E\Psi \left(r\right)$ with the potential (10) is reduced to the problem of a 2D harmonic oscillator with the exciton reduced mass μ. Following [49,50] one obtains the radial Schrödinger equation and the solution for the eigenfunctions for the relative motion of an electron and a hole in a GHAT3 double layer in terms of associated Laguerre polynomials, which can be written as

(11) $$\Psi_{NL}\left(r\right)=\frac{N!}{a^{|L|+1}\sqrt{\tilde{n}!{\tilde{n}}^{\prime }!}}2^{-|L|/2}\mathrm{sgn}{\left(L\right)}^{L}r^{\left|L\right|}e^{-r^{2}/\left(4a^{2}\right)}\times L_{N}^{\left|L\right|}\left(r^{2}/\left(2a^{2}\right)\right)\frac{e^{-iL\varphi }}{{\left(2\pi \right)}^{1/2}},$$

where $N=\min(\tilde{n},{\tilde{n}}^{\prime })$, $L=\tilde{n}-{\tilde{n}}^{\prime }$, $\tilde{n},$${\tilde{n}}^{\prime }=0,1,2,3,\ldots$ are the quantum numbers, φ is the polar angle, and $a={\left[\hbar /\left(2\sqrt{2\mu \gamma }\right)\right]}^{1/2}$ is a Bohr radius of a dipolar exciton. The corresponding energy spectrum is given by

(12) $$E_{NL}\equiv E_{e(h)}=-V_{0}+\left(2N+1+|L|\right)\hbar {\left(\frac{2\gamma }{\mu }\right)}^{1/2}.$$

At the lowest quantum state N=L=0 as it follows from Equation (12) the ground state energy for the exciton is given by

(13) $$E_{00}=-V_{0}+\hbar {\left(\frac{2\gamma }{\mu }\right)}^{1/2}.$$

The important characteristic of the exciton is the square of the in-plane gyration radius $r_{X}^{2}$. It allows one to estimate the condition when the excitonic gas is dilute enough. One can obtain the square of the in-plane gyration radius $r_{X}$ of a dipolar exciton [14], which is expressed as the average squared projection of an electron-hole separation onto the plane of a GHAT3 monolayer

(14) $$r_{X}^{2}\equiv \langle r^{2}\rangle =\int \Psi_{00}^{*}\left(r\right)r^{2}\Psi_{00}\left(r\right)d^{2}r=\frac{2\pi }{2\pi a^{2}}\int_{0}^{+\infty }r^{2}e^{-\frac{r^{2}}{2a^{2}}}rdr=2a^{2}.$$

We consider dipolar excitons, formed by an electron in the conduction band and a hole in the valence band (CV excitons) and formed by and electron in the conduction band and a hole in the intermediate valence band (CI excitons). For CV excitons one has

(15) $$\begin{array}{c} \mu_{CV}=\frac{m_{CB}m_{VB}}{m_{CB}+m_{VB}}=\frac{\Delta }{2v_{F}^{2}};\;M_{CV}=m_{CB}+m_{VB}=\frac{{\left(1+\alpha^{2}\right)}^{2}\Delta }{2v_{F}^{2}\alpha^{2}}\;. \end{array}$$

For CI excitons one has

(16) $$\begin{array}{c} \mu_{CI}=\frac{m_{CB}m_{IB}}{m_{CB}+m_{IB}}=\frac{\left(1+\alpha^{2}\right)\Delta }{2v_{F}^{2}\left(2-\alpha^{2}\right)};\;M_{CI}=m_{CB}+m_{IB}=\frac{\left(1+\alpha^{2}\right)\left(2-\alpha^{2}\right)\Delta }{2v_{F}^{2}\left(1-\alpha^{2}\right)}. \end{array}$$

## 3. The Collective Excitations Spectrum and Superfluidity for the Two-Component System of Dipolar Excitons

We consider the dilute limit for dipolar exciton gas in a GHAT3 double layer, when $n_{A}a_{B\;A}^{2}\ll 1$ and $n_{B}a_{B\;B}^{2}\ll 1$, where $n_{A(B)}$ and $a_{B\;A(B)}$ are the concentration and effective exciton Bohr radius for A(B) dipolar excitons, correspondingly. In the dilute limit, dipolar A and B excitons are formed by electron-hole pairs with the electrons and holes spatially separated in two different GHAT3 layers. We will treat the two-component weakly interacting Bose gas of dipolar excitons in a GHAT3 double layer by applying the approach analogous to the one used for dipolar excitons in a transition metal dichalcogenide (TMDC) double layer [16,17].

Since the dipolar excitons, formed by the charge carriers in different valleys, are characterized by the same energy, the exciton states are degenerate with respect to the valley degree of freedom. Therefore, we consider the Hamiltonian of the weakly interacting Bose gas of dipolar excitons, formed in a single valley. We will take into account the degeneracy of the exciton states with respect to spin and valley degrees of freedom by the introducing the spin and valley degeneracy factor s=16 below. The Hamiltonian $\hat{H}$ of the 2D A and B weakly interacting dipolar excitons can be written as

(17) $$\begin{array}{c} \hat{H}={\hat{H}}_{A}+{\hat{H}}_{B}+{\hat{H}}_{I}, \end{array}$$

where ${\hat{H}}_{A(B)}$ are the Hamiltonians of A(B) excitons defined as

(18) $$\begin{array}{c} {\hat{H}}_{A(B)}=\sum_{k}E_{A(B)}\left(k\right)a_{kA(B)}^{†}a_{kA(B)}+\frac{g_{AA(BB)}}{2S}\sum_{klm}a_{kA(B)}^{†}a_{lA(B)}^{†}a_{A(B)m}a_{A(B)k+l-m}, \end{array}$$

and ${\hat{H}}_{I}$ is the Hamiltonian of the interaction between A and B excitons presented as

(19) $$\begin{array}{c} {\hat{H}}_{I}=\frac{g_{AB}}{S}\sum_{klm}a_{kA}^{†}a_{lB}^{†}a_{Bm}a_{Ak+l-m}, \end{array}$$

where $a_{kA(B)}^{†}$ and $a_{kA(B)}$ are Bose creation and annihilation operators for A(B) dipolar excitons with the wave vector k, correspondingly, *S* is the area of the system, $E_{A(B)}\left(k\right)\equiv \epsilon_{A(B)}=\varepsilon_{\left(0)A(B\right)}\left(k\right)+A_{A(B)}$ is the energy spectrum of non-interacting A(B) dipolar excitons, respectively, $\varepsilon_{\left(0)A(B\right)}\left(k\right)=\hbar^{2}k^{2}/\left(2M_{A(B)}\right)$, $M_{A(B)}$ is an effective mass of non-interacting dipolar excitons, $A_{A(B)}$ is the constant, which depends on A(B) dipolar exciton binding energy and the corresponding gap, $g_{AA(BB)}$ and $g_{AB}$ are the interaction constants for the repulsion between two A dipolar excitons, two B dipolar excitons and for the interaction between A and B dipolar excitons, respectively.

In dilute system with large interlayer separation *D*, two dipolar excitons, located at distance *R*, repel each other via the dipole-dipole interaction potential $U\left(R\right)=\kappa e^{2}D^{2}/\left(\epsilon_{d}R^{3}\right)$. Following the procedure described in [51], the interaction parameters for the exciton-exciton repulsion in very dilute systems can be obtained implying the exciton-exciton dipole-dipole repulsion exists only at the distances between excitons greater than the distance from the exciton to the classical turning point.

The many-particle Hamiltonian for a weakly interacting Bose gas can be diagonalized within the Bogoliubov approximation [52], replacing the product of four operators in the interaction term with the product of two operators. The Bogoliubov approximation is valid if one assumes that most of the particles belong to BEC. In this case, in the Hamiltonian one can keep only the terms responsible for the interactions between the condensate and non-condensate particles, while the terms describing the interactions between non-condensate particles are neglected.

Following the procedure, described in [16,17], applying the Bogoliubov approximation [52], generalized for a two-component weakly interacting Bose gas [53,54] and introducing the following notation,

(20) $$\begin{array}{ccc} G_{AA} & = & g_{AA}n_{A}=gn_{A},\;G_{BB}=g_{BB}n_{B}=gn_{B},\;G_{AB}=g_{AB}\sqrt{n_{A}n_{B}}=g\sqrt{n_{A}n_{B}},\\ \omega_{A}\left(k\right) & = & \sqrt{\varepsilon_{(0)A}^{2}\left(k\right)+2G_{AA}\varepsilon_{(0)A}\left(k\right)},\\ \omega_{B}\left(k\right) & = & \sqrt{\varepsilon_{(0)B}^{2}\left(k\right)+2G_{BB}\varepsilon_{(0)B}\left(k\right)}, \end{array}$$

one obtains two modes of the spectrum of Bose collective excitations $\varepsilon_{j}\left(k\right)$

(21) $$\begin{array}{c} \varepsilon_{j}\left(k\right)=\sqrt{\frac{\omega_{A}^{2}\left(k\right)+\omega_{B}^{2}\left(k\right)+{\left(-1\right)}^{j-1}\sqrt{{\left(\omega_{A}^{2}\left(k\right)-\omega_{B}^{2}\left(k\right)\right)}^{2}+{\left(4G_{AB}\right)}^{2}\varepsilon_{(0)A}\left(k\right)\varepsilon_{(0)B}\left(k\right)}}{2}}, \end{array}$$

where j=1, 2. In our approach, the condition $G_{AB}^{2}=G_{AA}G_{BB}$ holds.

At small momenta p=ħk, when $\varepsilon_{(0)A}\left(k\right)\ll G_{AA}$ and $\varepsilon_{(0)B}\left(k\right)\ll G_{BB}$, expanding the spectrum of collective excitations $\varepsilon_{j}\left(k\right)$ up to the first order with respect to the momentum *p*, one obtains two sound modes in the spectrum of the collective excitations $\varepsilon_{j}\left(p\right)=c_{j}p$, where $c_{j}$ is the sound velocity written as

(22) $$\begin{array}{c} c_{j}=\sqrt{\frac{G_{AA}}{2M_{A}}+\frac{G_{BB}}{2M_{B}}+{\left(-1\right)}^{j-1}\sqrt{{\left(\frac{G_{AA}}{2M_{A}}-\frac{G_{BB}}{2M_{B}}\right)}^{2}+\frac{G_{AB}^{2}}{M_{A}M_{B}}}}, \end{array}$$

At j=1, the spectrum of collective excitations is determined by the non-zero sound velocity $c_{1}$, while at j=2 the sound velocity vanishes with $c_{2}=0$. At large momenta, for the conditions when $\varepsilon_{(0)A}\left(k\right)\gg G_{AA}$ and $\varepsilon_{(0)B}\left(k\right)\gg G_{BB}$, one obtains two parabolic modes of collective excitations with the spectra $\varepsilon_{1}\left(k\right)=\varepsilon_{(0)A}\left(k\right)$ and $\varepsilon_{2}\left(k\right)=\varepsilon_{(0)B}\left(k\right)$, if $M_{A}<M_{B}$ and if $M_{A}>M_{B}$ with the spectra $\varepsilon_{1}\left(k\right)=\varepsilon_{(0)B}\left(k\right)$ and $\varepsilon_{2}\left(k\right)=\varepsilon_{(0)A}\left(k\right)$.

## 4. Superfluidity of the Weakly-Interacting Bose Gas of Dipolar Excitons

Since when j=2 the sound velocity vanishes, below we take into account only the branch of the spectrum of collective excitations at j=1, neglecting the branch at j=2. According to [52,55], it is clear that we need a finite sound velocity for superfluidity. Since the branch of the collective excitations at zero sound velocity for the collective excitations corresponds to the zero energy of the quasiparticles (which means that no quasiparticles are created with zero sound velocity), this branch does not lead to the dissipation of energy resulting in finite viscosity and, therefore, does not influence the Landau critical velocity. This is the reason for eliminating the zero sound velocity case in our considerations here. The weakly-interacting gas of dipolar excitons in the double layer of GHAT3 satisfies the Landau criterion for superfluidity [52,55], because at small momenta, the energy spectrum of the quasiparticles in the weakly-interacting gas of dipolar excitons at j=1 is sound-like with the finite sound velocity, $c_{1}$. In the moving weakly-interacting gas of dipolar excitons the quasiparticles are created at velocities above the velocity of sound, and the critical velocity for superfluidity reads as $v_{c}=c_{1}$. The difference between the ideal Bose gas and two-component weakly interacting Bose gas of dipolar excitons is that while the spectrum of ideal Bose gas has no branch with finite sound velocity, the dipolar exciton system under consideration has one branch in the spectrum of collective excitations with finite sound velocity at j=1 due to exciton-exciton interaction. Therefore, at low temperatures, the two-component system of dipolar excitons exhibits superfluidity due to exciton-exciton interactions, while the ideal Bose gas does not demonstrate superfluidity.

We defined the density of the superfluid component $\rho_{s}\left(T\right)$ as $\rho_{s}\left(T\right)=\rho -\rho_{n}\left(T\right)$, where $\rho =M_{A}n_{A}+M_{B}n_{B}$ is the total 2D density of the dipolar excitons and $\rho_{n}\left(T\right)$ denotes the density of the normal component. The density $\rho_{n}\left(T\right)$ of the normal component can be defined using standard procedure [56]. The assumption that the dipolar exciton system moves with a velocity u implies that the superfluid component moves with the velocity u. The energy dissipation at nonzero temperatures *T* is characterized by the occupancy of quasiparticles in this system. Since the density of quasiparticles is small at low temperatures, the gas of quasiparticles can be treated as an ideal Bose gas. In order to obtain the density of the superfluid component, one can define the total mass flow for a Bose gas of quasiparticles in the frame, in which the superfluid component is assumed to be at rest, as

(23) $$\begin{array}{c} J=s\int \frac{d^{2}p}{{\left(2\pi \hbar \right)}^{2}}pf\left[\varepsilon_{1}\left(p\right)-p\cdot u\right], \end{array}$$

where s=16 is the spin and valley degeneracy factor, $f\left[\varepsilon_{1}\left(p\right))\right]={\left(\exp\left[\varepsilon_{1}\left(p\right)/\left(k_{B}T\right)\right]-1\right)}^{-1}$ is the Bose-Einstein distribution function for the quasiparticles with the dispersion $\varepsilon_{1}\left(p\right)$, and $k_{B}$ is the Boltzmann constant. Expanding the expression under the integral in Equation (23) up to the first order with respect to $p\cdot u/(k_{B}T)$, one has:

(24) $$\begin{array}{c} J=-s\frac{u}{2}\int \frac{d^{2}p}{{\left(2\pi \hbar \right)}^{2}}p^{2}\frac{\partial f\left[\varepsilon_{1}\left(p\right)\right]}{\partial \varepsilon_{1}\left(p\right)}\;. \end{array}$$

The density $\rho_{n}$ of the normal component in the moving weakly-interacting Bose gas of dipolar excitons is defined as [56]

(25) $$\begin{array}{c} J=\rho_{n}u\;. \end{array}$$

Employing Equations (24) and (25), one derives the normal component density as

(26) $$\begin{array}{c} \rho_{n}\left(T\right)=-\frac{s}{2}\int \frac{d^{2}p}{{\left(2\pi \hbar \right)}^{2}}p^{2}\frac{\partial f\left[\varepsilon_{1}\left(p\right)\right]}{\partial \varepsilon_{1}\left(p\right)}\;. \end{array}$$

At low temperatures $k_{B}T\ll M_{A(B)}c_{j}^{2}$, the small momenta ($\varepsilon_{(0)A}\left(k\right)\ll G_{AA}$ and $\varepsilon_{(0)B}\left(k\right)\ll G_{BB}$) make the dominant contribution to the integral on the right-hand side of Equation (26). The quasiparticles with such small momenta are characterized by the sound spectrum $\varepsilon_{1}\left(k\right)=c_{1}k$ with the sound velocity defined by Equation (22). By substituting $\varepsilon_{1}\left(k\right)=c_{1}k$ into Equation (26), we obtain

(27) $$\begin{array}{c} \rho_{n}\left(T\right)=\frac{3s\zeta (3)}{2\pi \hbar^{2}c_{1}^{4}}k_{B}^{3}T^{3}, \end{array}$$

where ζ(z) is the Riemann zeta function (ζ(3)≃1.202).

The mean field critical temperature $T_{c}$ of the phase transition at which the superfluidity occurs, implying neglecting the interaction between the quasiparticles, is obtained from the condition $\rho_{s}\left(T_{c}\right)=0$ [56]:

(28) $$\begin{array}{c} \rho_{n}\left(T_{c}\right)=\rho =M_{A}n_{A}+M_{B}n_{B}\;. \end{array}$$

At low temperatures $k_{B}T\ll M_{A(B)}c_{1}^{2}$ by substituting Equation (27) into Equation (28), one derives

(29) $$\begin{array}{c} T_{c}={\left[\frac{2\pi \hbar^{2}\rho c_{1}^{4}}{3\zeta \left(3\right)sk_{B}^{3}}\right]}^{1/3}\;. \end{array}$$

While Bose-Einstein condensation occurs at absolute zero even in a two-dimensional (2D) system, it is well known that in a 2D bosonic system, Bose-Einstein condensation does not occur at finite temperature, and only the quasi-long-range order appears. In this paper, we have obtained the mean-field critical temperature $T_{c}$ of the phase transition at which superfluidity appears without claiming BEC in a 2D system at finite temperature. In this work, we have considered BEC only at absolute zero temperature. The similar approach has been applied for excitons in TMDC double layers [16,17].

## 5. Discussion

**Figure 2:**
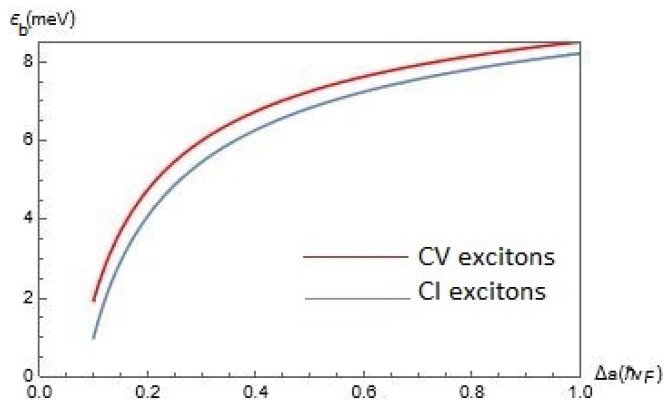
The exciton binding energy $E_{b}\left(\alpha ,\Delta ,D\right)$ for CV and CI excitons as functions of the gap Δ for chosen parameter α=0.6 and interlayer separations D=25nm. The lattice constant of $\alpha -T_{3}$ is a=2.46.

In this section we now discuss the results of our calculations. In Figure 2, we present the results for the exciton binding energy $E_{b}\left(\alpha ,\Delta ,D\right)$ for CV and CI excitons as functions of the gap Δ for chosen parameter α=0.6 and interlayer separations D=25nm. According to Figure 2, $E_{b}\left(\alpha ,\Delta ,D\right)$ is an increasing function of Δ, whereas for CV excitons the exciton binding energy is slightly larger than that for CI excitons.

**Figure 3:**
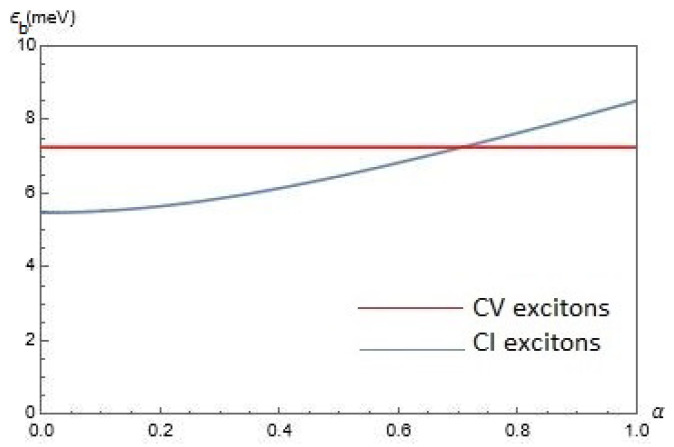
The exciton binding energy $E_{b}\left(\alpha ,\Delta ,D\right)$ for CV and CI excitons as functions of the parameter α for chosen gap $\Delta =0.5\;\hbar v_{F}/a$ and interlayer separations D=25nm.

In Figure 3, we present our results for the exciton binding energy $E_{b}\left(\alpha ,\Delta ,D\right)$ for CV and CI excitons as functions of the parameter α for chosen gap $\Delta =0.5\;\hbar v_{F}/a$ and interlayer separations D=25nm. According to Figure 3, $E_{b}\left(\alpha ,\Delta ,D\right)$ does not depend on α for CV excitons, whereas it is an increasing function of α for CI excitons. At α≲0.7$E_{b}\left(\alpha ,\Delta ,D\right)$ for CV excitons is larger than for CI excitons, while at α≳0.7$E_{b}\left(\alpha ,\Delta ,D\right)$ for CI excitons is larger than for CV excitons.

**Figure 4:**
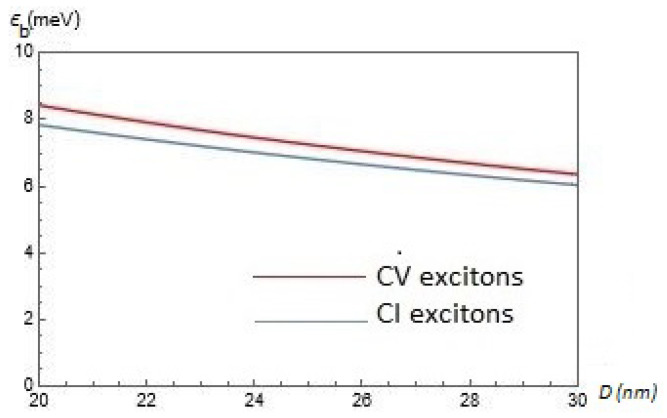
The exciton binding energy $E_{b}\left(\alpha ,\Delta ,D\right)$ for CV and CI excitons as functions of the interlayer separation *D* for chosen parameter α=0.6 and gap $\Delta =0.5\;\hbar v_{F}/a$.

In Figure 4, we present the results of our calculations for the exciton binding energy $E_{b}\left(\alpha ,\Delta ,D\right)$ for CV and CI excitons as functions of the interlayer separation *D* for chosen parameter α=0.6 and gap $\Delta =0.5\;\hbar v_{F}/a$. According to Figure 4, $E_{b}\left(\alpha ,\Delta ,D\right)$ is a decreasing function of *D*, whereas for CV excitons the exciton binding energy is slightly larger than for CI excitons.

**Figure 5:**
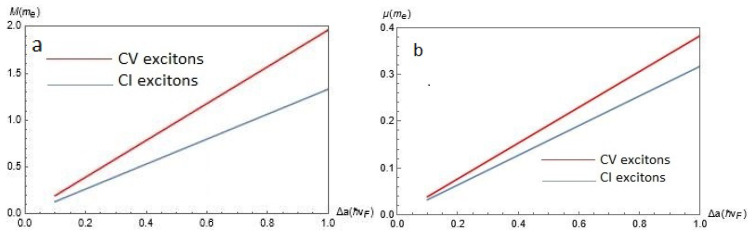
The effective masses of a dipolar exciton for CV and CI excitons as functions of the gap Δ for chosen α=0.6 for (**a**) center-of-mass exciton mass *M* on the left panel and (**b**) reduced exciton mass μ, on the right.

In Figure 5, we present plots of the effective masses for CV and CI dipolar excitons as functions of the gap Δ for chosen α=0.6 for (a) center-of-mass exciton mass *M* on the left-hand side and (b) reduced exciton mass μ, on the right. According to Figure 5, both *M* and μ for the CV and CI excitons are increasing functions of Δ, while for CV excitons both *M* and μ are slightly larger than for CI excitons.

**Figure 6:**
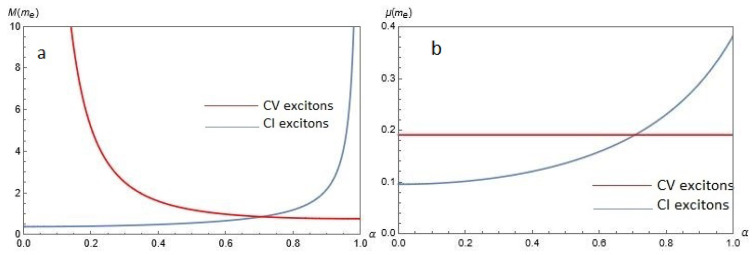
The effective masses of dipolar excitons for CV and CI excitons as functions of the hopping parameter α for chosen gap $\Delta =0.5\;\hbar v_{F}/a$ for (**a**) center-of-mass exciton mass *M* in the left panel and (**b**) reduced exciton mass μ, on the right.

Figure 6 shows the effective masses of a dipolar exciton for CV and CI excitons as functions of α for chosen $\Delta =0.5\;\hbar v_{F}/a$ for (a) center-of-mass exciton mass *M* in the left panel and (b) reduced exciton mass μ, on the right. According to Figure 6, for CV excitons *M* is a decreasing function of α, whereas μ does not depend on α. For CI excitons, both *M* and μ increase as α is increased. For α≲0.7, both *M* and μ for CV excitons are larger than for CI excitons, but when α≳0.7$E_{b}\left(\alpha ,\Delta ,D\right)$ both *M* and μ for CV excitons are smaller than for CI excitons.

**Figure 7:**
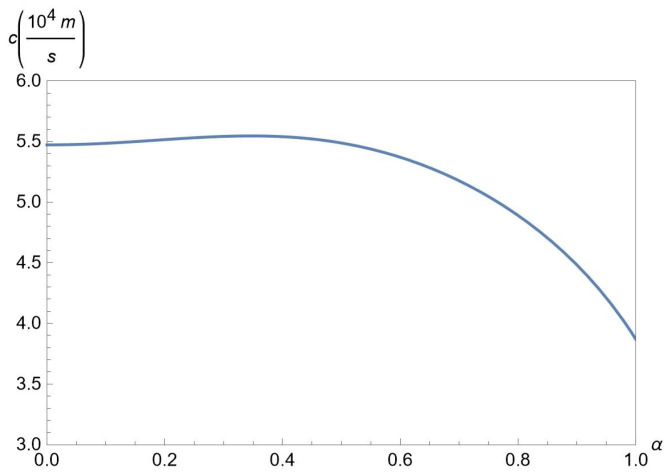
Plot of the sound velocity $c\equiv c_{1}$ versus α for chosen gap $\Delta =\hbar v_{F}/a$, interlayer separations D=25nm at fixed concentrations $n_{A}=50\times 10^{11}\;{\mathrm{cm}}^{-2}$ and $n_{B}=50\times 10^{11}\;{\mathrm{cm}}^{-2}$ of A and B excitons, respectively.

Figure 7 demonstrates the dependence of the sound velocity $c\equiv c_{1}$ on the hopping parameter α for chosen $\Delta =\hbar v_{F}/a$, interlayer separations D=25nm at fixed concentrations $n_{A}=50\times 10^{11}\;{\mathrm{cm}}^{-2}$ and $n_{B}=50\times 10^{11}\;{\mathrm{cm}}^{-2}$ of A and B excitons, respectively. According to Figure 7, *c* does not depend much on α when α≲0.5, while for α≳0.5, the sound velocity *c* is a decreasing function of α.

**Figure 8:**
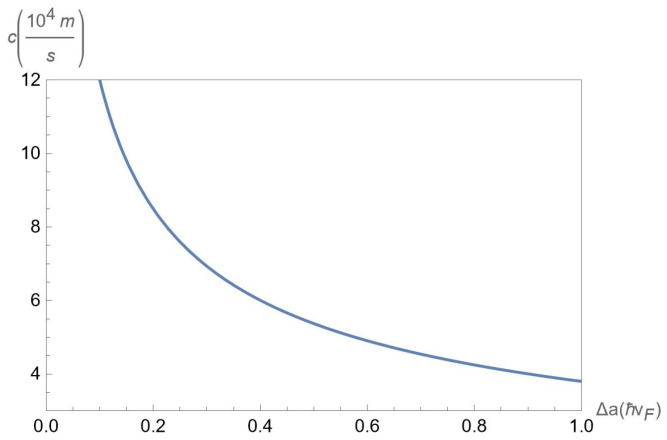
The sound velocity $c\equiv c_{1}$ versus the gap Δ for chosen parameter α = 0.6, interlayer separations D=25nm at the fixed concentrations $n_{A}=50\times 10^{11}\;{\mathrm{cm}}^{-2}$ and $n_{B}=50\times 10^{11}\;{\mathrm{cm}}^{-2}$ of A and B excitons, respectively.

In Figure 8, we plot the sound velocity $c\equiv c_{1}$ versus the gap Δ for chosen parameter α=0.6, interlayer separations D=25nm for chosen concentrations $n_{A}=50\times 10^{11}\;{\mathrm{cm}}^{-2}$ and $n_{B}=50\times 10^{11}\;{\mathrm{cm}}^{-2}$ of A and B excitons, respectively. According to Figure 8, the sound velocity *c* is a decreasing function of Δ.

**Figure 9:**
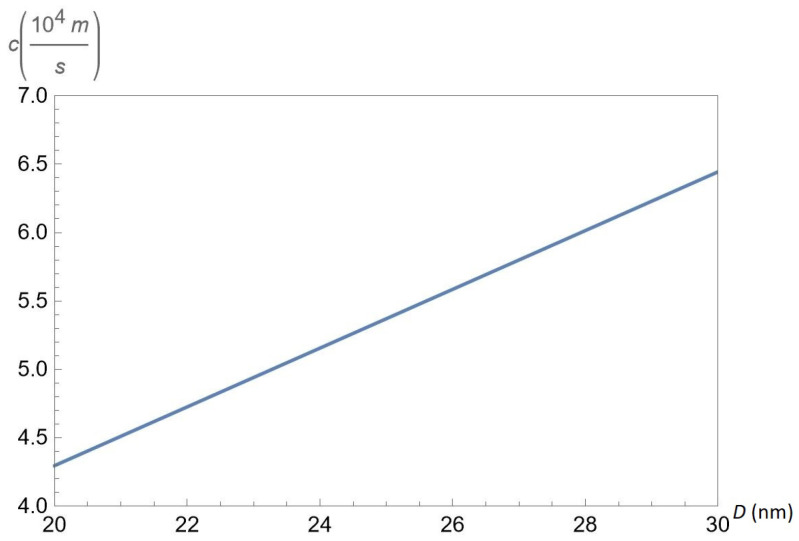
The sound velocity $c\equiv c_{1}$ versus the interlayer separation *D* for chosen parameter α=0.6 and gap $\Delta =0.5\;\hbar v_{F}/a$, at fixed concentrations $n_{A}=50\times 10^{11}\;{\mathrm{cm}}^{-2}$ and $n_{B}=50\times 10^{11}\;{\mathrm{cm}}^{-2}$ of A and B excitons, respectively.

In Figure 9, we show the sound velocity $c\equiv c_{1}$ as a function of the interlayer separation *D* for hopping parameter α=0.6 and gap $\Delta =0.5\;\hbar v_{F}/a$, for fixed concentrations $n_{A}=50\times 10^{11}\;{\mathrm{cm}}^{-2}$ and $n_{B}=50\times 10^{11}\;{\mathrm{cm}}^{-2}$ of A and B excitons, respectively. According to Figure 9, the sound velocity *c* is an increasing function of *D*.

**Figure 10:**
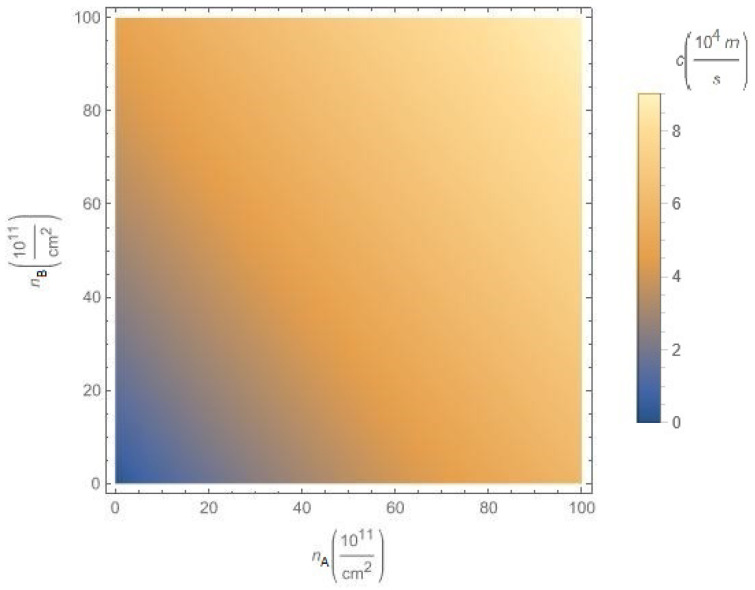
The sound velocity $c\equiv c_{1}$ versus the concentrations $n_{A}$ and $n_{B}$ of A and B excitons, respectively, for chosen parameter α=0.6 and gap $\Delta =0.5\;\hbar v_{F}/a$, at the fixed interlayer separation D=25nm.

In Figure 10, we illustrate the dependence of the sound velocity $c\equiv c_{1}$ on the concentrations $n_{A}$ and $n_{B}$ of A and B excitons, respectively for chosen hopping parameter α=0.6 and gap $\Delta =0.5\;\hbar v_{F}/a$, at fixed interlayer separation D=25nm. According to Figure 10, the sound velocity *c* is an increasing function of both concentrations $n_{A}$ and $n_{B}$.

**Figure 11:**
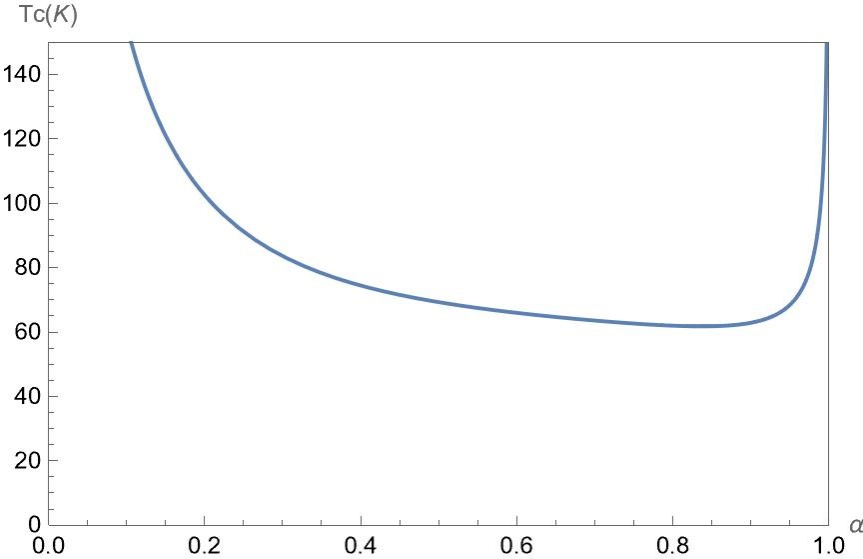
The mean-field phase transition critical temperature $T_{c}\left(n_{A},n_{B},\alpha ,\Delta ,D\right)$ versus the parameter α for chosen gap $\Delta =0.5\;\hbar v_{F}/a$, interlayer separations D=25nm at the fixed concentrations $n_{A}=50\times 10^{11}\;{\mathrm{cm}}^{-2}$ and $n_{B}=50\times 10^{11}\;{\mathrm{cm}}^{-2}$ of A and B excitons, respectively.

In Figure 11, we present the mean-field phase transition critical temperature $T_{c}\left(n_{A},n_{B},\alpha ,\Delta ,D\right)$ as a function of the parameter α for chosen gap $\Delta =0.5\;\hbar v_{F}/a$, interlayer separations D=25nm at the fixed concentrations $n_{A}=50\times 10^{11}\;{\mathrm{cm}}^{-2}$ and $n_{B}=50\times 10^{11}\;{\mathrm{cm}}^{-2}$ of A and B excitons, respectively. According to Figure 11, $T_{c}$ is a decreasing function of α at α≲0.9, while at α≳0.9 the critical temperature $T_{c}$ is an increasing function of α.

**Figure 12:**
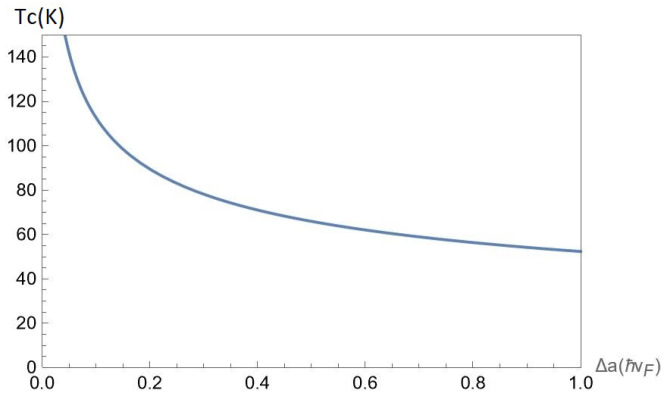
The mean-field phase transition critical temperature $T_{c}\left(n_{A},n_{B},\alpha ,\Delta ,D\right)$ versus the gap Δ for chosen parameter α=0.6, interlayer separations D=25nm at the fixed concentrations $n_{A}=50\times 10^{11}\;{\mathrm{cm}}^{-2}$ and $n_{B}=50\times 10^{11}\;{\mathrm{cm}}^{-2}$ of A and B excitons, respectively.

In Figure 12, we present the mean-field phase transition critical temperature $T_{c}\left(n_{A},n_{B},\alpha ,\Delta ,D\right)$ as a function of the gap Δ for chosen parameter α=0.6, interlayer separations D=25nm at the fixed concentrations $n_{A}=50\times 10^{11}\;{\mathrm{cm}}^{-2}$ and $n_{B}=50\times 10^{11}\;{\mathrm{cm}}^{-2}$ of A and B excitons, respectively. According to Figure 12, the criticaltemperature $T_{c}$ is a decreasing function of Δ.

**Figure 13:**
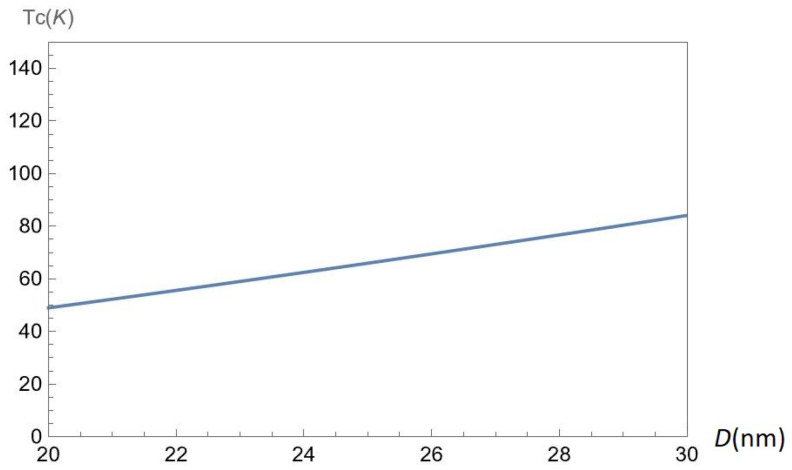
The mean-field phase transition critical temperature $T_{c}\left(n_{A},n_{B},\alpha ,\Delta ,D\right)$ versus the interlayer separation *D* for chosen parameter α=0.6 and gap $\Delta =0.5\;\hbar v_{F}/a$, at fixed concentrations $n_{A}=50\times 10^{11}\;{\mathrm{cm}}^{-2}$ and $n_{B}=50\times 10^{11}\;{\mathrm{cm}}^{-2}$ of A and B excitons, respectively.

In Figure 13, we demonstrate the mean-field phase transition critical temperature $T_{c}\left(n_{A},n_{B},\alpha ,\Delta ,D\right)$ as a function of the interlayer separation *D* for chosen parameter α=0.6 and gap $\Delta =0.5\;\hbar v_{F}/a$, at the fixed concentrations $n_{A}=50\times 10^{11}\;{\mathrm{cm}}^{-2}$ and $n_{B}=50\times 10^{11}\;{\mathrm{cm}}^{-2}$ of A and B excitons, respectively. According to Figure 13, the critical temperature $T_{c}$ is an increasing function of *D*.

**Figure 14:**
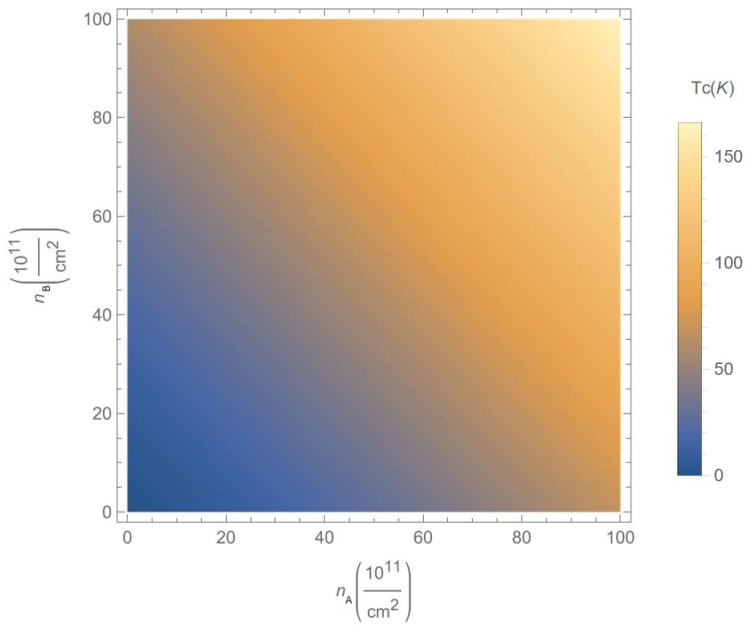
Density plot for the mean-field phase transition critical temperature $T_{c}\left(n_{A},n_{B},\alpha ,\Delta ,D\right)$ versus the concentrations $n_{A}$ and $n_{B}$ of A and B excitons, respectively, for chosen parameter α=0.6 and gap $\Delta =0.5\;\hbar v_{F}/a$, at the fixed interlayer separation D=25nm.

In Figure 14, we present density plots for the mean-field phase transition critical temperature $T_{c}\left(n_{A},n_{B},\alpha ,\Delta ,D\right)$ as a function of the concentrations $n_{A}$ and $n_{B}$ of A and B excitons, respectively for chosen parameter α=0.6 and gap $\Delta =0.5\;\hbar v_{F}/a$, at the fixed interlayer separation D=25nm. According to Figure 14, the the critical temperature $T_{c}$ is an increasing function of both the concentrations $n_{A}$ and $n_{B}$.

At a formal level, the weakly interacting Bose gas of A and B dipolar excitons in a GHAT3 double layer are similar to the two-component weakly interacting Bose gas of trapped cold atoms in a planar harmonic trap. The spectrum of collective excitations in the Bogoliubov approximation for dipolar excitons in a GHAT3 double layer is similar to one for a two-component BEC of trapped cold atoms, studied in [53,54].

The gap parameter Δ, has a dual role, since it appears as chemical potential in the Hamiltonian, as also in the mass of the excitons through the band curvature. According to Figure 2 and Figure 12, the dipolar exciton binding energy is an increasing function of the gap Δ, while is the mean-field phase transition temperature $T_{c}$ is a decreasing function of the gap Δ. Therefore, there should be an optimal value for Δ, which would correspond to relatively high $T_{c}$ at the relatively high dipolar exciton binding energy. The latter condition provides the formation of the superfluid phase by the relatively stable dipolar excitons.

Note that electron-hole superfluids can be formed not only in the BEC regime but also in the BCS-BEC crossover regime [7]. Quantum Monte Carlo simulations analyzing the BCS-BEC crossover regime for electron-hole systems have been performed [57]. In this paper, we concentrate on the dilute electron-hole system, which corresponds to the BEC, which matches experimentally achievable densities in the electron-hole systems in 2D materials. BCS regime requires higher concentrations beyond the model of weakly interacting Bose gas. The studies of the BCS regime, and BEC-BCS crossover for an electron-hole superfluid in a GHAT3 double layer, seem to be a promising direction for future studies.

The considered system of dipolar excitons in a GHAT3 double layer has also a strong similarity, with photon condensation in a cavity. The collective modes and possibility of the Kosterlitz-Thouless phase transition to the superfluid phase [58,59] has been studied for a photon condensation in a cavity in [60]. If we consider only one type of excitons in a GHAT3 double layer, assuming the concentration of the excitons of another type to be zero, the expressions for the spectrum of collective excitations reported in this paper can be reduced to the expressions similar to [60].

The Kosterlitz-Thouless phase transition to the superfluid phase [58,59] can be inferred from the variation of the superfluid density, which has been computed in this paper.

Note that, in this paper, we did not consider vortices, as within the mean-field approximation it was assumed that the number of quasiparticles are relatively low. However, beyond the mean-field approximation, it is possible to consider the properties of vortices in the system of dipolar excitons. Thus, the dynamical creation of fractionalized vortices and vortex lattices can be considered by applying the approach, developed for the BEC of cold atoms in [61].

The Josephson phenomena for two trapped condensates of dipolar excitons can be studied by applying an approach similar to the one developed for non-Abelian Josephson effect between two F=2 spinor Bose-Einstein condensates of cold atoms in double optical traps [62].

## 6. Conclusions

This paper is devoted to an investigation of the existence of BEC and superfluidity of dipolar excitons in double layers of GHAT3 which was proposed and analyzed. We have derived the solution of a two-body problem for an electron and a hole for the model Hamiltonian representing double-layer GHAT3. We predict the formation of two types of dipolar excitons, characterized by different binding energies and effective masses, in the double layer of GHAT3. We have calculated the binding energy, effective mass, spectrum of collective excitations, superfluid density, and the mean-field critical temperature of the phase transition to the superfluid state for the two-component weakly interacting Bose gas of A and B dipolar excitons in double-layer GHAT3. We have demonstrated that at fixed exciton density, the mean-field critical temperature for superfluidity of dipolar excitons is decreased as a function of the gap Δ. Our results show that $T_{c}$ is increased as a function of the density *n* and is decreased as a function of the gap Δ and the interlayer separation *D*.

The occupancy of the superfluid state at $T<T_{c}$ can result in the existence of persistent dissipationless superconducting oppositely directed electric currents in each GHAT3 layer, forming a double layer. According to the presented results of our calculations, while the external weak magnetic field, responsible for the formation of the gap Δ in the double layer of $\alpha -T_{3}$ increases the exciton binding energy, the mean-field transition temperature to the superfluid phase is increased as the weak magnetic field and Δ are decreased. Therefore, the dipolar exciton system in a double-layer of GHAT3 can be applied to engineer a switch, where transport properties of dipolar excitons can be tuned by an external weak magnetic field, forming the gap Δ. Varying a weak magnetic field may lead to a phase transition between the superfluid and normal phase, which sufficiently changes the transport properties of dipolar excitons.

## Data Availability

The data presented in this study are available on request from the corresponding author.
